# Diabetes Management by Fourth-Generation Glucose Sensors Based on Lemon-Extract-Supported CuO Nanoporous Materials

**DOI:** 10.3390/molecules28196763

**Published:** 2023-09-22

**Authors:** Israr U. Hassan

**Affiliations:** Department of Mathematics & Sciences, College of Arts & Applied Sciences, Dhofar University, Salalah PC 211, Oman; i_hassan@du.edu.om

**Keywords:** glucose sensing, nanocomposite, non-enzymatic, CuO@lemon extract

## Abstract

Diabetes is a major worldwide health issue, impacting millions of people around the globe and putting pressure on healthcare systems. Accurate detection of glucose is critical for efficient diabetes care, because it allows for prompt action to control blood sugar levels and avoid problems. Reliable glucose-sensing devices provide individuals with real-time information, allowing them to make more educated food, medicine, and lifestyle decisions. The progress of glucose sensing holds the key to increasing the quality of life for diabetics and lowering the burden of this prevalent condition. The present investigation addresses the synthesis of a CuO@lemon-extract nanoporous material using the sol–gel process. Scanning electron microscopy (SEM) and transmission electron microscopy (TEM) were used to analyze the morphological properties of the composite, which revealed a homogeneous integration of CuO nanoparticles (NPs) on the surface of the matrix. The existence of primarily oxidized copper species, especially CuO, was confirmed by X-ray diffraction spectroscopy (XRD) investigation in combination with energy-dispersive X-ray (EDX) spectroscopy. The CuO@lemon-extract-modified glassy carbon electrode (CuO@lemon-extract GCE) performed well in non-enzymatic electrochemical sensing applications such as differential pulse voltammetry (DPV) and amperometric glucose detection. The electrode achieved a notable sensitivity of 3293 µA mM^−1^ cm^−2^ after careful adjustment, with a noticeable detection limit of 0.01 µM (signal-to-noise ratio of 3). The operational range of the electrode was 0.01 µM to 0.2 µM, with potential applied of 0.53 V vs. Ag/AgCl. These findings underscore the CuO@lemon-extract GCE’s promise as a robust and reliable platform for electrochemical glucose sensing, promising advances in non-enzymatic glucose sensing (NEGS) techniques.

## 1. Introduction

The rise of rapid, accurate, and affordable technologies for glucose sensing has sparked great interest in the field of biosensor research in recent years. According to the World Health Organization (WHO), over 537 million persons had diabetes in 2022, accounting for approximately 8.5% of the total adult population [1]. This illness can lead to a variety of consequences, including heart attacks, strokes, renal failure, decreased eyesight, and nerve damage [2,3]. Monitoring glucose levels is critical in diabetes care, sparking a search for novel sensing technologies with enhanced accuracy and usability. Enzymatic sensors for glucose have long dominated the industry because of their high specificity and sensitivity; nevertheless, their high production costs and low stability have pushed researchers to investigate other alternatives [4,5]. Non-enzymatic glucose sensing (NEGS) is emerging as an exciting possibility in this area, with the potential for increased durability, cheaper production costs, and less interference from diverse chemicals [6,7]. Transition metal oxides have risen to prominence among NEGS materials due to their exceptional catalytic characteristics and electron transport capabilities [8].

Metal and metal oxide nanomaterials’ unusual electrical, mechanical, and catalytic capabilities have captivated substantial interest for many years [8,9]. The nanoparticles of CuO are a well-known example of this, with uses in a wide range of industries as a result of their low toxicity, high conductivity, ecologically acceptable nature, and inexpensive cost [9]. Biosensors, antifouling products coatings, plasmonics, amination coupling processes, antibacterial paints, food packaging, and medication delivery are among the uses [10]. Copper-based materials for sensing in different shapes and topologies, as, for example, nanosheets, microspheres, hollow spheres, nanowires, and hollow polyhedrons, have received substantial scientific attention [11,12,13,14,15]. These materials have increased surface areas and active spots for NEGS, which can be very beneficial [16].

In the field of glucose sensing, significant progress has been made through the use of several copper oxide (CuO) nanostructures, each with unique sensing properties. Wang et al. [17] used electrospinning to create CuO nanofibers that were then placed onto an ITO substrate. This strategic approach resulted in a remarkable sensitivity of 431.3 µA mM^−1^ cm^−2^, which might have been due to the huge particular surface area and 3D design following immobilization. Zhang et al. [18] pursued the synthesis of CuO nanowires (NWs) in a two-step wet chemical method, reaching a significant glucose sensitivity of 648.2 mA mM^−1^ cm^−2^. Wang et al. [19] extended the investigation by introducing flower-like CuO nanostructures, resulting in a 709.53 µA mM^−1^ cm^−2^ glucose detection sensitivity. Ni et al. [20] pioneered a novel methodology by directly growing CuO NWs on a copper foil, utilizing a low-cost, template-free solution-based process. A glucose sensor with a sensitivity of 1886.3 A mM^−1^ cm^−2^ and a LOD of 0.05 mM has been developed as a result of this breakthrough. These various approaches show how useful nanoporous CuO is for identifying the presence of glucose.

In the current work, we introduce a novel NEGS method that makes use of a composite material made of copper oxide nanoparticles (CuO) and lemon extract. This novel combination is an attractive choice for a variety of biological applications due to its fascinating features which improve sensor performance. The production of CuO nanoporous material uses lemon extract, which is rich in bioactive substances, including flavonoids and polyphenols, as a stabilizing and capping agent. To emphasize the relevance of our approach, it is crucial to highlight the benefits of the green method, as used in our study, above conventional approaches. Comparing the green approach used in this study to conventional procedures, there are a number of clear benefits. First, by removing the need for dangerous chemicals and reducing waste production, it has a big, positive influence on the environment. The green technique is also more economical because it uses less energy and materials. Furthermore, since chemical reagent contamination is often eliminated, it frequently produces goods of superior quality. It adheres to sustainable and environmentally friendly concepts which are gaining significance in today’s research environment [21,22]. These benefits not only advance the discipline of green chemistry as a whole but also highlight the usefulness of our technique. These nanoparticles’ well-defined morphological and structural characteristics make them an ideal platform for glucose sensing. This research effort’s main goal is to assess the CuO@lemon-extract composite’s electrochemical performance, sensitivity, selectivity, and stability as a glucose sensor. We predict that by combining cutting-edge materials and bioactive components, our NEGS technique will pave the way for the creation of next-generation, cost-effective, and dependable glucose monitoring systems. Such developments might have a considerable influence on diabetes care and overall quality of life, while also broadening the horizon for other biological uses.

## 2. Results and Discussion

### 2.1. XRD and EDX Analysis

**Figure 1:**
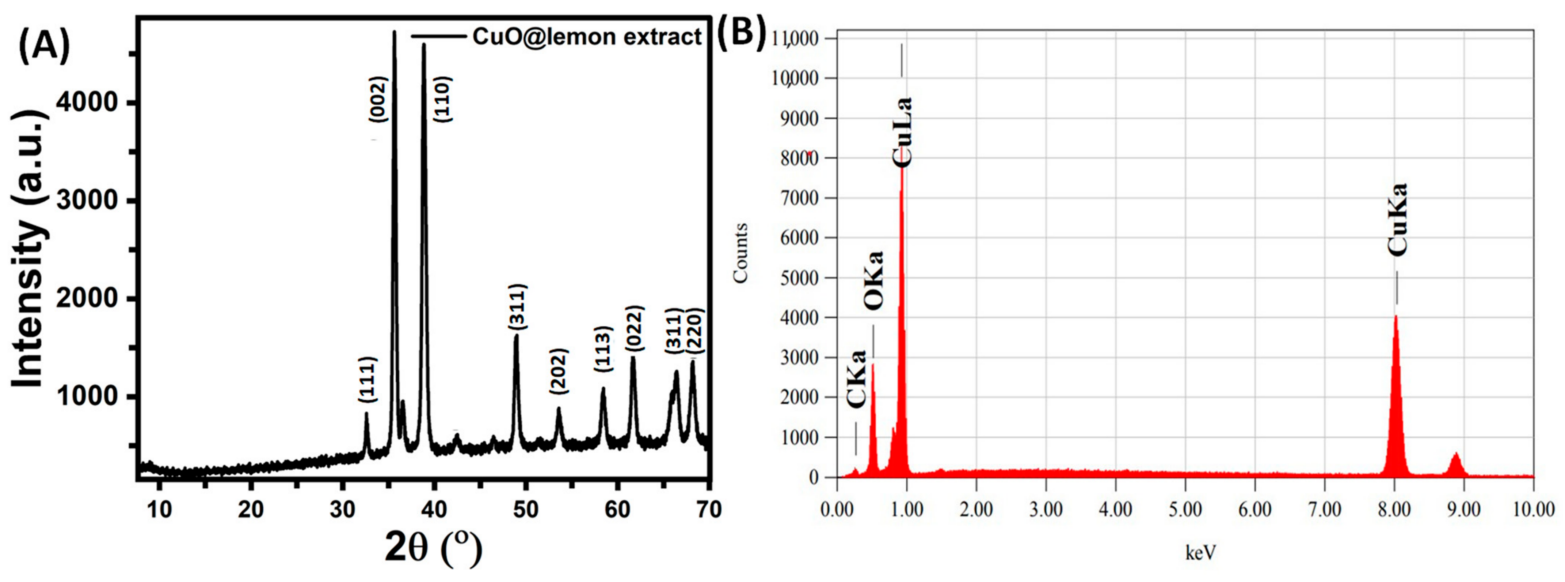
(**A**) XRD spectra of CuO@lemon extract and (**B**) EDX spectrum for CuO@lemon extract.

The XRD analysis revealed distinct diffraction peaks at 2θ values of 32.3°, 35.6°, 38.8°, 48.5°, 53.5°, 59.8°, 61.4°, 66.2°, and 68.2° which can be attributed to the (111), (002), (110), (311), (202), (113), (022), (311) and (220) crystal planes of monoclinic CuO, respectively, as shown in Figure 1A. These characteristic peaks indicate the formation of highly crystalline CuO nanoparticles within the composite material. The absence of any other diffraction peaks in the XRD pattern suggests the high purity of the CuO@lemon-extract nanoporous materials, with no significant impurities or other phases present. Furthermore, the XRD pattern reveals that the synthesized CuO nanoparticles possess a well-defined crystalline structure, which is indicative of their excellent stability and potential for various applications. The presence of the lemon extract in the composite may have contributed to the formation of a nanoporous structure, as indicated by broader diffraction peaks compared to pure CuO. In Figure 1B, the EDX analysis confirmed the presence of two main elements in the nanocomposite. The prominent peaks at energy levels of approximately 1.0 keV and 8.0 keV correspond, respectively, to the characteristic Lα and Kα X-ray emissions of copper (Cu). This peak array validates the successful incorporation of CuO nanoparticles within the nanocomposite, consistent with the intended synthesis. Another peak at an energy level of around 0.5 keV corresponds to the Kα X-ray emission of oxygen (O). This confirms the presence of oxygen atoms in the CuO@lemon-extract nanocomposite, which is essential for the formation of copper oxide (CuO). Additionally, the EDX spectrum indicates the presence of carbon (C) peaks originating from the organic components of the lemon extract which act as stabilizing and capping agents during the nanocomposite synthesis.

### 2.2. SEM and TEM Analysis

**Figure 2:**
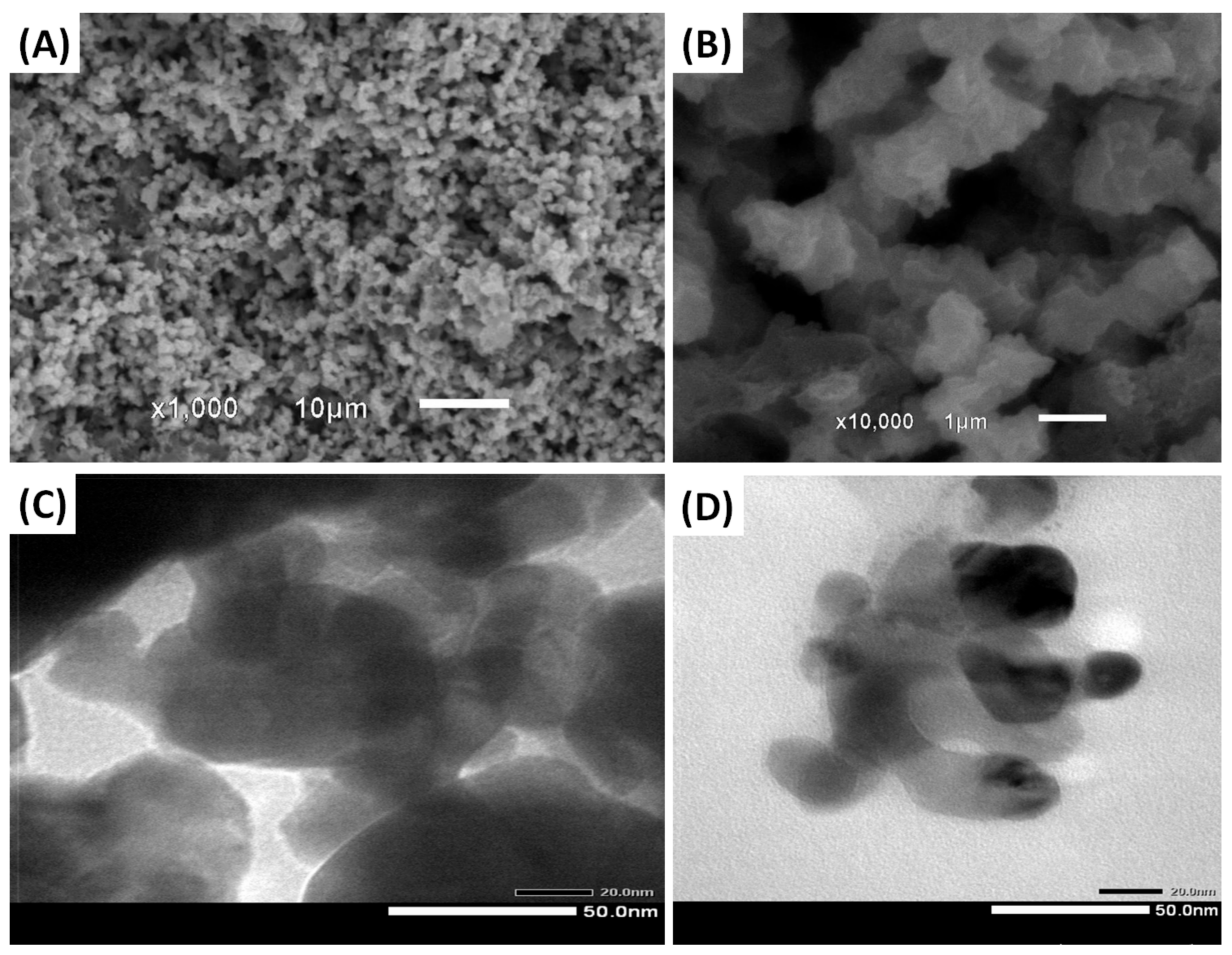
SEM results of (**A**) CuO@lemon extract at 10 µm scale and (**B**) CuO@lemon extract at 1 µm scale; TEM images of (**C**) CuO@lemon extract at 50.0 nm and (**D**) CuO@lemon extract at 50.0 nm, with a smaller particle size.

As seen in the SEM image in Figure 2A, the CuO@lemon-extract nanocomposite exhibits a highly porous and interconnected structure at 10 µm scale. The nanoporous morphology is visible as a network of irregularly shaped voids and channels dispersed uniformly throughout the material. This nanoporous structure is attributed to the incorporation of lemon extract during the synthesis process, which might have led to the formation of pores or voids due to the removal of certain components during the drying or calcination steps. The nanoparticles of copper oxide (CuO) are also evident in the more focused SEM image, Figure 2B, dispersed within the porous matrix of the lemon extract at 1 µm scale. These nanoparticles seem to be evenly dispersed and agglomerate into tiny clusters, both of which contribute to the overall nanoporous structure. The existence of well-defined CuO nanoparticles in the CuO@lemon-extract nanoporous materials, together with their observed nanoporous structure, implies that the CuO@lemon-extract nanoporous materials may have increased surface areas and better accessibility to target analytes. Individual nanoparticles are clearly apparent as black, well-defined entities scattered among a brighter backdrop in Figure 2C, which corresponds to the lemon-extract matrix. Ranging from 10 to 20 nm in size, the nanoparticles have a rather consistent size distribution. This size consistency suggests a controlled synthesis process, likely influenced by the presence of the lemon extract, which may have acted as a template or stabilizing agent during nanoparticle formation. The TEM image at 50 nm magnification (Figure 2D) provides a detailed view of the CuO@lemon-extract nanocomposite, showcasing the well-defined nanoparticles embedded within the lemon-extract matrix. The uniform size distribution, crystalline nature, and interactions between nanoparticles and lemon-extract components further validate the successful synthesis of this nanocomposite.

## 3. Electrochemical Study

### 3.1. Electrode-Analyte Kinetic Parameters Evaluation

**Figure 3:**
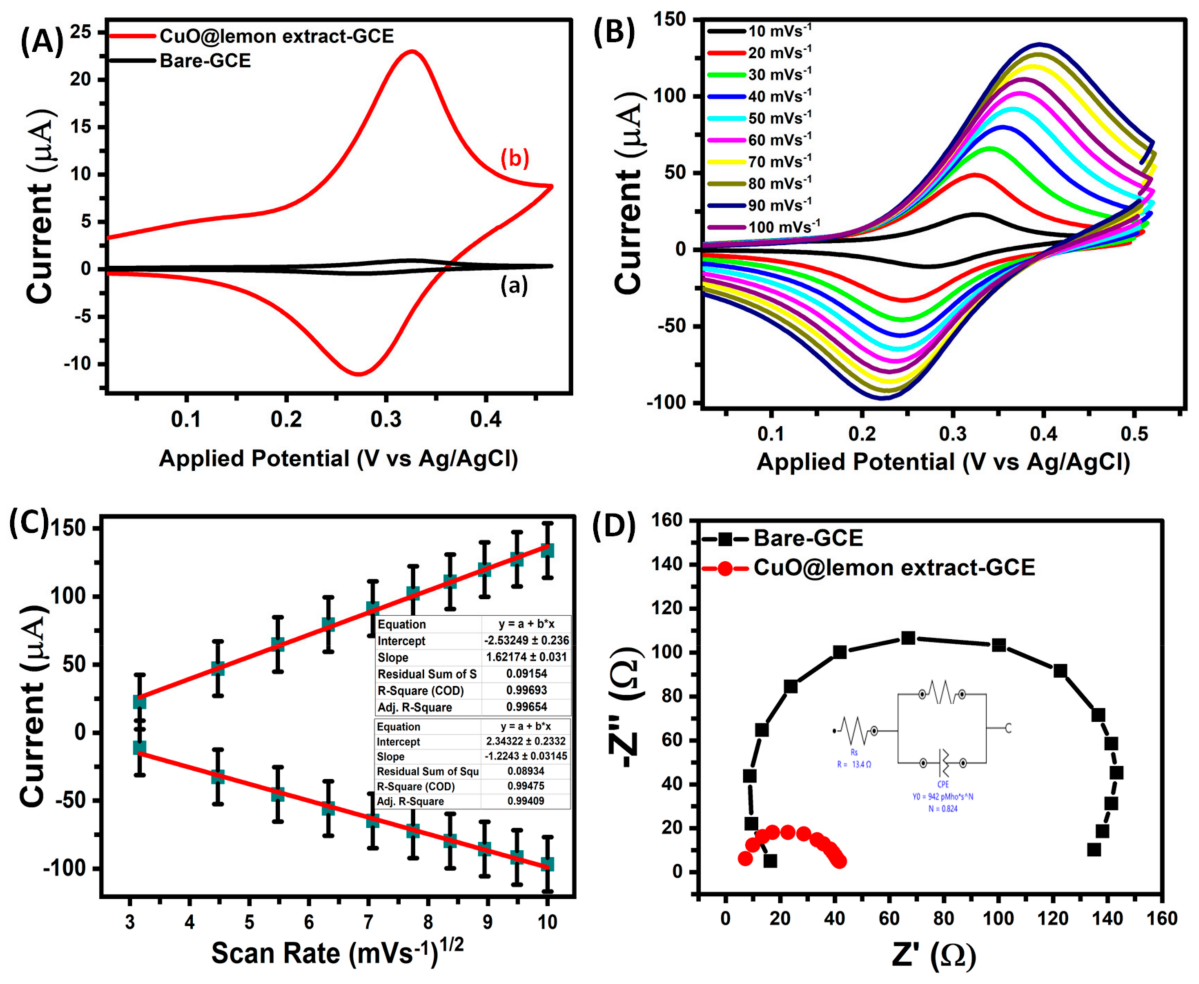
(**A**) CV study for (**b**) CuO@lemon-extract GCE at 10 to 100 mVs^−1^ in the presence of a 1.0 mM K_3_Fe(CN)_6_ probe in a 0.1 M KCl electrolyte, ((**a**) Bare-GCE) (**B**) CuO@lemon-extract GCE at different scan rates, (**C**) plot of peak current vs. square root of scan rate, and (**D**) Nyquist plot of CuO@lemon-extract GCE at frequency range of 0.1 Hz to 10^6^ Hz in the presence of a 1.0 mM K_3_Fe(CN)_6_ probe in a 0.1 M KCl electrolyte.

Both unmodified and modified glassy carbon electrodes (GCE) were used as working electrodes in a three-electrode configuration with a platinum wire and silver/silver chloride performing as the counter and reference electrodes, respectively. CV and electrochemical impedance spectroscopy (EIS) were used to analyze the surfaces of these electrodes. Figure 3A shows cyclic voltammograms for a 1.0 mM K_3_Fe(CN)_6_ probe in a 0.1 M KCl solution using bare GCE, and CuO@lemon-extract GCE at a scan rate of 10 mVs^−1^.

(1) $$I_{pa}=0.4463{\left(\frac{F^{3}}{RT}\right)}^{1/2}n^{3/2}A_{0}D_{0}^{1/2}Cv^{1/2}$$

where $I_{pa}$ is anodic peak current, *A*_0_ is electroactive surface area of electrode (cm^2^), R is molar gas constant (8.314 JK^−1^ mol^−1^), F is Faraday’s constant (96,540 C mol^−1^), $D_{0}\;$is diffusion coefficient (1.0 mM concentration C of Fe(CN)_6_^3−/4−^), and T is Kelvin temperature. The electrode surface area was derived from (Equation (1)); the slope of the $I_{pa}$ versus *v*^1/2^ plot (Figure 3C) was obtained from cyclic voltammetric measurements of different scan rate graphs at 10 to 100 mVs^−1^ (Figure 3B) of the 1.0 mM K_3_Fe(CN)_6_ probe in a 0.1 M KCl electrolyte at various scan speeds using the Randles–Sevick equation [22]. The electrochemical surface areas of unmodified GCE and CuO@lemon-extract GCE were calculated to be 0.030 cm^2^, and 1.920 cm^2^, respectively.

The EIS study (Figure 3D) was performed using the Potentiostat-Galvanostat Metrohm 302 N throughout a frequency range of 0.1 Hz to 10^6^ Hz while keeping a fixed amplitude of 10.0 mV. The goal was to examine the electrode materials’ Faradaic resistance and capacitive properties. The Randles-like equivalent circuit used for impedance analysis is depicted in the inset in Figure 3D. The reported equivalence series resistance values for bare-GCE and CuO@lemon-extract GCE, respectively, were 65.1 Ω and 26.2 Ω. CuO@lemon-extract GCE’s smaller equivalent series resistance value, which aligns almost parallel to the *Y*-axis at lower frequencies, suggests its advantageous conductive and capacitive characteristics, emphasizing efficient ion transport inside the electrode.

**Figure 4:**
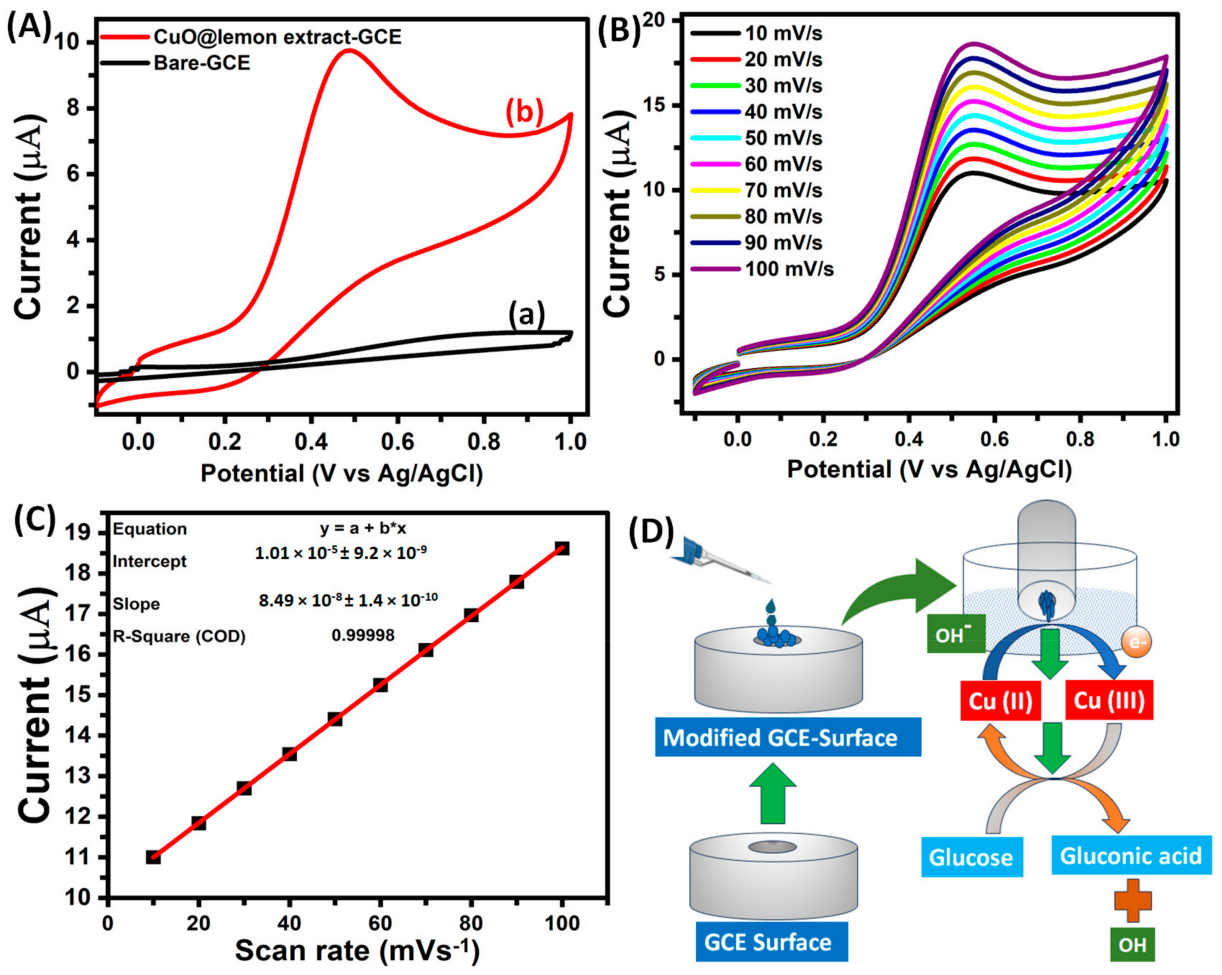
(**A**) CV record of (**a**) bare GCE and (**b**) CuO@lemon-extract GCE in presence of 0.1 mM glucose with 0.1 M NaOH at the various electrodes (scan rate, 10 mVs^−1^); (**B**) with the same solution of glucose CV recorded of CuO@lemon-extract GCE in 0.1 mM glucose at various scan rates (10–100 mVs^−1^); (**C**) calibration between current vs. the different scan rate plot; and (**D**) possible illustration of glucose to gluconic acid.

Modified nanoporous GCEs exhibit electrocatalytic activity in glucose sensing due to their robust ability for electro-oxidation and low-overpotential electron-transfer mechanisms. This is accompanied by their affordability and stability. To evaluate their electrochemical performance, CV was conducted in 0.1 M NaOH with a scan rate of 10 mV/s, as depicted in Figure 4A. CVs using both bare-GCE and CuO@lemon-extract-modified GCE sensors displayed negligible redox responses, indicating limited functionality under alkaline conditions. However, upon utilizing modified electrodes incorporating CuO and lemon extract (as illustrated in Figure 4A(a),(b)), distinct and well-defined peaks emerged. The chemical reaction occurring at the electrode surface can be described as follows:

(2) $$CuO+2OH¯\to \;{Cu\;(OH)}_{2}+2e¯$$

(3) $${Cu\left(OH\right)}_{2}+\;{OH}^{-}\to \;CuO\;\left(OH\right)+{2H}_{2}O+e¯$$

As demonstrated in Figure 4C, the CuO@lemon-extract GCE sensor displays a notably higher $I_{pa}$ value of 18.6 μA at −0.5 V compared to Ag/AgCl when using a scan rate of 100 mV/s. This process takes place at elevated potentials, resulting in an amplified current at the peak. The heightened current is indicative of the successful glucose oxidation on the CuO@lemon-extract electrode. The effect of scan rate upon the oxidation of glucose response curve was also examined utilizing 0.1 mM glucose with a 0.1 M NaOH the solution, as shown in Figure 4B. This observation signifies that the oxidation of glucose is a controlled process occurring on the electrode’s surface. The oxidative current exhibited a direct relationship with the scan rate, demonstrating a strong correlation, with a value of 0.999. Figure 4D shows a possible illustration of the transformation of glucose to gluconic acid.

**Figure 5:**
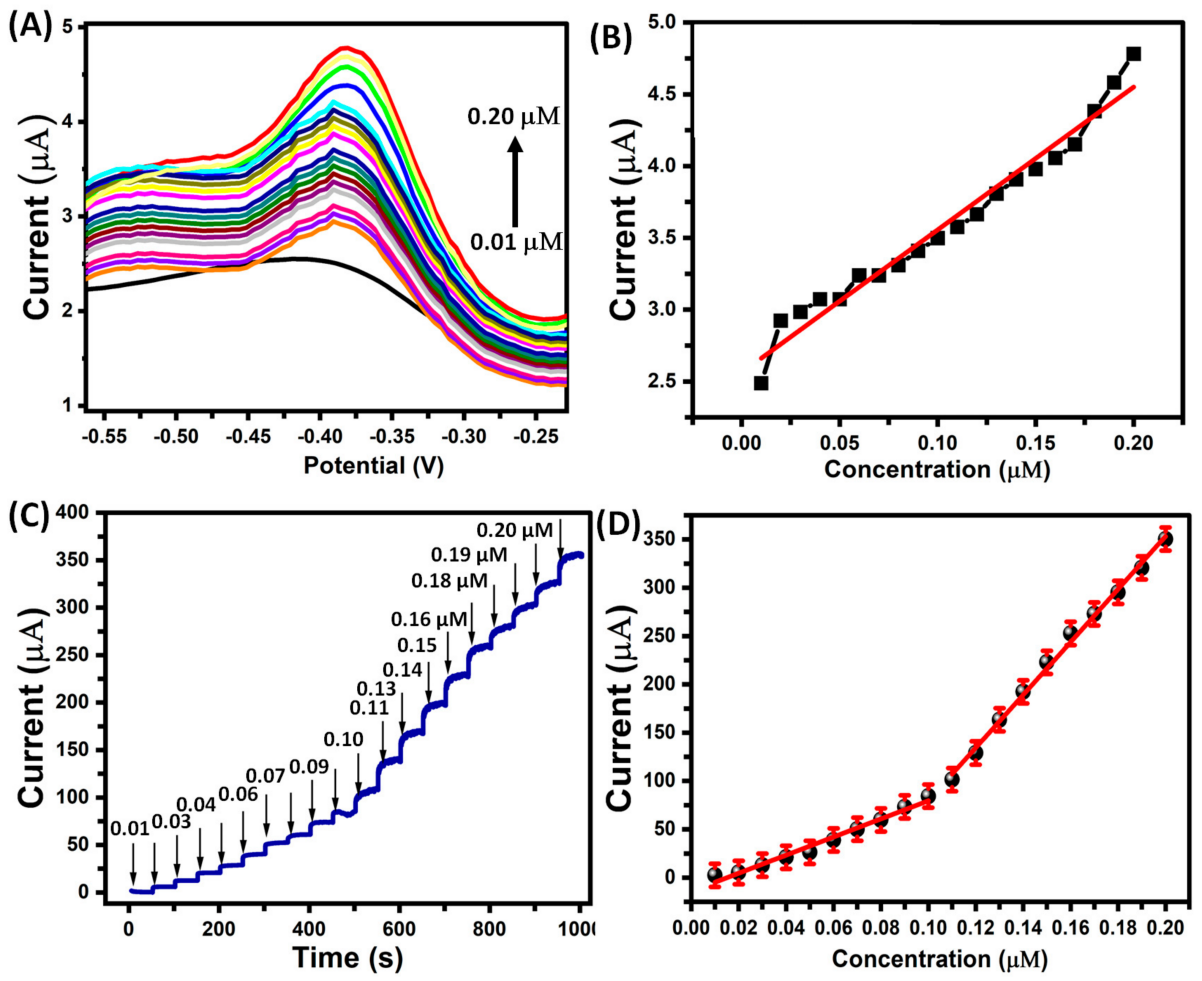
(**A**) DPV response of CuO@lemon-extract GCE towards 0.01 μM to 0.20 μM glucose with 0.1 M NaOH. (**B**) The calibration plot of current intensity vs. concentration (μM). (**C**) Amperometry response of CuO@lemon-extract GCE towards 0.01 µM and 0.2 µM glucose with 0.1 M NaOH. (**D**) The calibration plot of current intensity vs. concentration (μM).

The incorporation of a glucose solution in the alkaline environment enhances glucose oxidation via the CuO@lemon-extract GCE sensor, resulting in an enhanced current, owing to the electron transfer represented in Figure 5A. This causes a considerable increase in the peak. This enhancement can be attributed to the robust interaction between CuO and lemon extract. The incorporation of lemon extract further augments the effective surface area, creating highly active sites for glucose oxidation. Moreover, the CuO@lemon-extract GCE surface promotes rapid glucose diffusion and adsorption, along with facilitating electron transport. The substantial current increase is a consequence of the conversion of glucose molecules into gluconolactone. In comparison to unmodified electrodes, nanoporous CuO@lemon-extract GCE exhibits superior electrochemical activity for glucose oxidation. Notably, when compared to bare electrodes or CuO@lemon-extract GCE, the CuO@lemon-extract GCE demonstrates enhanced electrochemical performance in glucose oxidation. The chemical response governing glucose oxidation is as follows at the electrode interface:

(4) $$glucose\;\to gluconolactone+e^{-}$$

(5) $$Cu\left(III\right)+e^{-}\;\to Cu(II)$$

(6) $$Cu\left(III\right)+glucose+e^{-}\to gluconolactone+Cu(II)$$

(7) Gluconolactone→ Gluconic acid (hydrolysis)

The electrocatalytic activity of the CuO@lemon-extract-modified GCE in response to glucose is studied further using amperometric current–time measurements after adding different glucose concentrations sequentially. Figure 5C depicts the modified electrode’s amperometric response at an applied potential of 0.53 V vs. Ag/AgCl when 0.01 M and 0.2 M glucose are consecutively added into an alkaline solution (0.1 M NaOH). The oxidation current exhibited a gradual rise and stabilized within approximately 15 s. The corresponding calibration plot (Figure 5B) demonstrates a linear relationship (R^2^ = 0.998) across a concentration range of 0.01 µM to 0.10 µM, with a slope expressed as Ip = 937.29 − 14.153 × [glucose] (µM). Upon close examination of lower concentrations of glucose and considering that current noise is 0.1 µA cm^−2^, a LOD was achieved for 0.01 µM at a signal-to-noise ratio (S/N) of 3. This LOD is on par with those reported for other NEGS techniques (refer to Table 1) [6,23,24,25,26,27,28,29,30,31].

**Figure 6:**
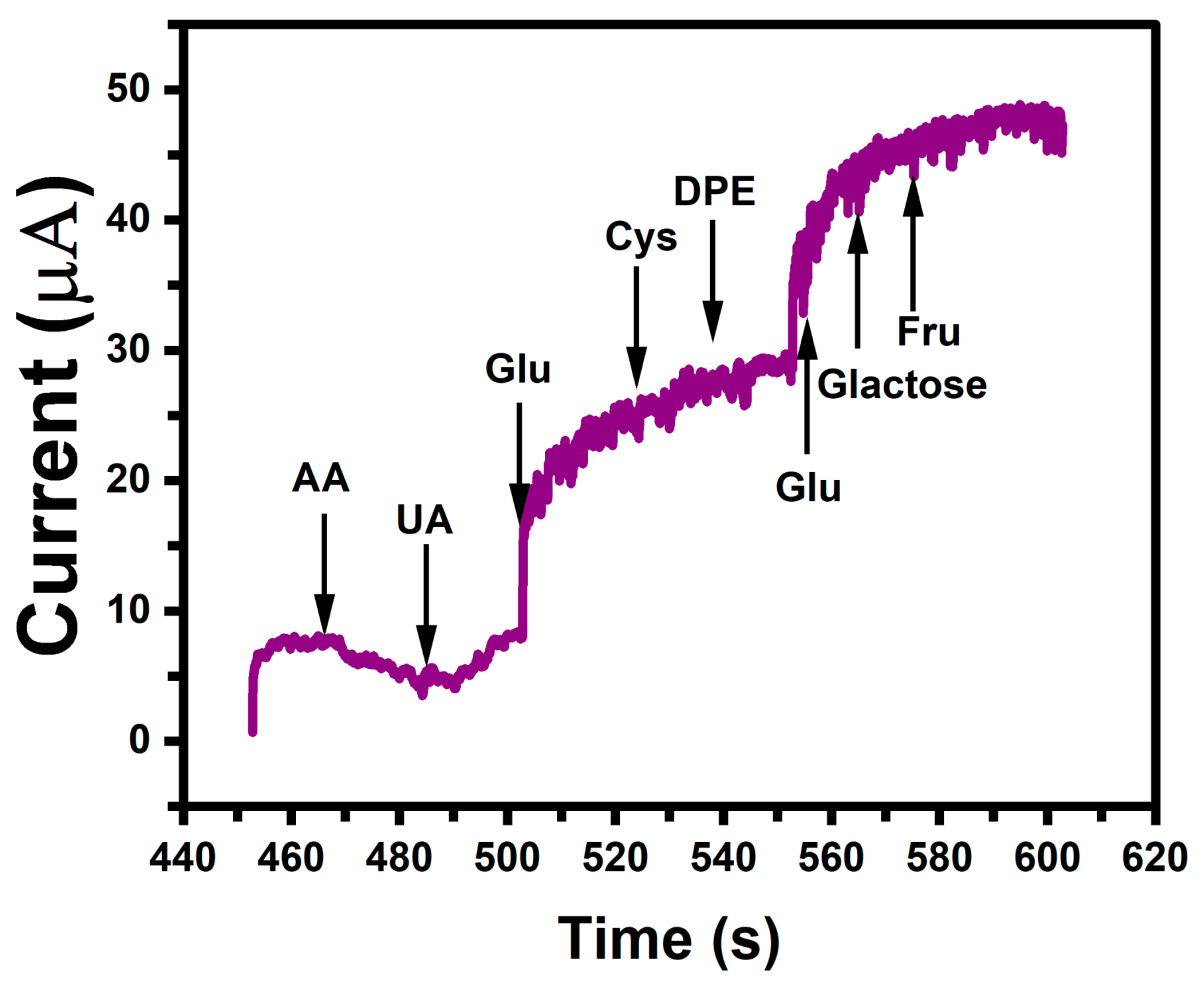
A study of the effects of several interfering compounds.

The presence of multiple substances, such as ascorbic acid (AA), uric acid (UA), dopamine (DPE), cysteine (Cys), and other carbohydrates like fructose (Fru) or galactose, might possibly impact the detection selectivity for determining glucose, especially at higher levels of glucose. As shown in Figure 6A, the response of the glucose (Glu) current is unaltered even when exposed to 10-times higher amounts of AA, UA, Cys, and DPE. This underscores the exceptional sensor selectivity.

Furthermore, the electrode reacts only to glucose, with no catalytic currents found for other carbohydrates derivatives such as fructose or galactose. The RSD, which exhibits a value of 4.9% for a concentration of glucose of 0.1 mM, quantifies the repeatability of electrode production and its utilization in sensing of glucose. The functioning of the electrode was assessed after a two-month period of storage in a refrigerator at 4 °C to determine long-term stability. At a tested potential of −0.53 V vs. Ag/AgCl, the sensor retained approximately 97% of its original current response when exposed to 0.1 mM glucose when immersed in 0.1 M NaOH aqueous solution, indicating the electrode’s impressive durability.

### 3.2. Mechanism of CuO@lemon-Extract GCE for Glucose Oxidation

The specific mechanism of glucose oxidation on the CuO modified electrode in an alkaline environment is unknown. However, in electrocatalysis, efficient binding at the electrode–electrolyte interface, as well as fast electron mobility inside the electrode, play critical roles. At present, Marioli and Kuwana’s suggested model for glucose oxidation on copper-based modified electrodes implies a series of steps [32]. This involves deprotonation of glucose to produce enediol, adsorption onto the electrode surface, and oxidation aided by Cu(II) and Cu(III) species. Glucose oxidation is shown to occur within a potential range of 0.40–0.80 V, corresponding with the Cu(II)/Cu(III) oxidation wave. It is proposed that Cu(III) species act as electron transfer mediators. Cu(II) on the CuO electrode is initially oxidized to Cu(III), which subsequently functions as a catalyst for glucose oxidation during CV measurements [33,34,35,36]. This process produces gluconolactone, which is then converted into gluconic acid. The Cu(II)/Cu(III) redox pair facilitates the electrochemical non-enzymatic detection of glucose oxidation, as shown in Figure 4C. Notably, the oxidation process may involve more than just gluconic acid generation, with C-C bond breakage possibly providing lighter compounds like formates and carbonates that might engage in electron transfer [37]. As a result, in the presence of a glucose solution, the CuO modified electrode is expected to produce an increased catalytic current response, with the amplitude of the response current corresponding to glucose concentration (see Figure 4A).

### 3.3. Tests for Reproducibility, Reusability, and Stability

The amperometric measurements of glucose at +0.53 V were examined utilizing four altered sensor electrodes made under comparable conditions, allowing the repeatability of the findings as to the CuO@lemon-extract GCE sensors to be assessed. The same electrode was used to record 1000 additional consecutive amperograms. The RSD of the CuO@lemon-extract GCE sensor was less than 4.9%, indicating outstanding reproducibility and repeatability. After a month of usage, the electrode’s stability was evaluated. The CuO@lemon-extract GCE sensor retained 96% of its original current responses, indicating good stability. During these testing, the monolith was securely secured to the GCE surface. Before repeating, it was continually washed and electrochemically energized.

## 4. Experimental Section

**Scheme 1:**
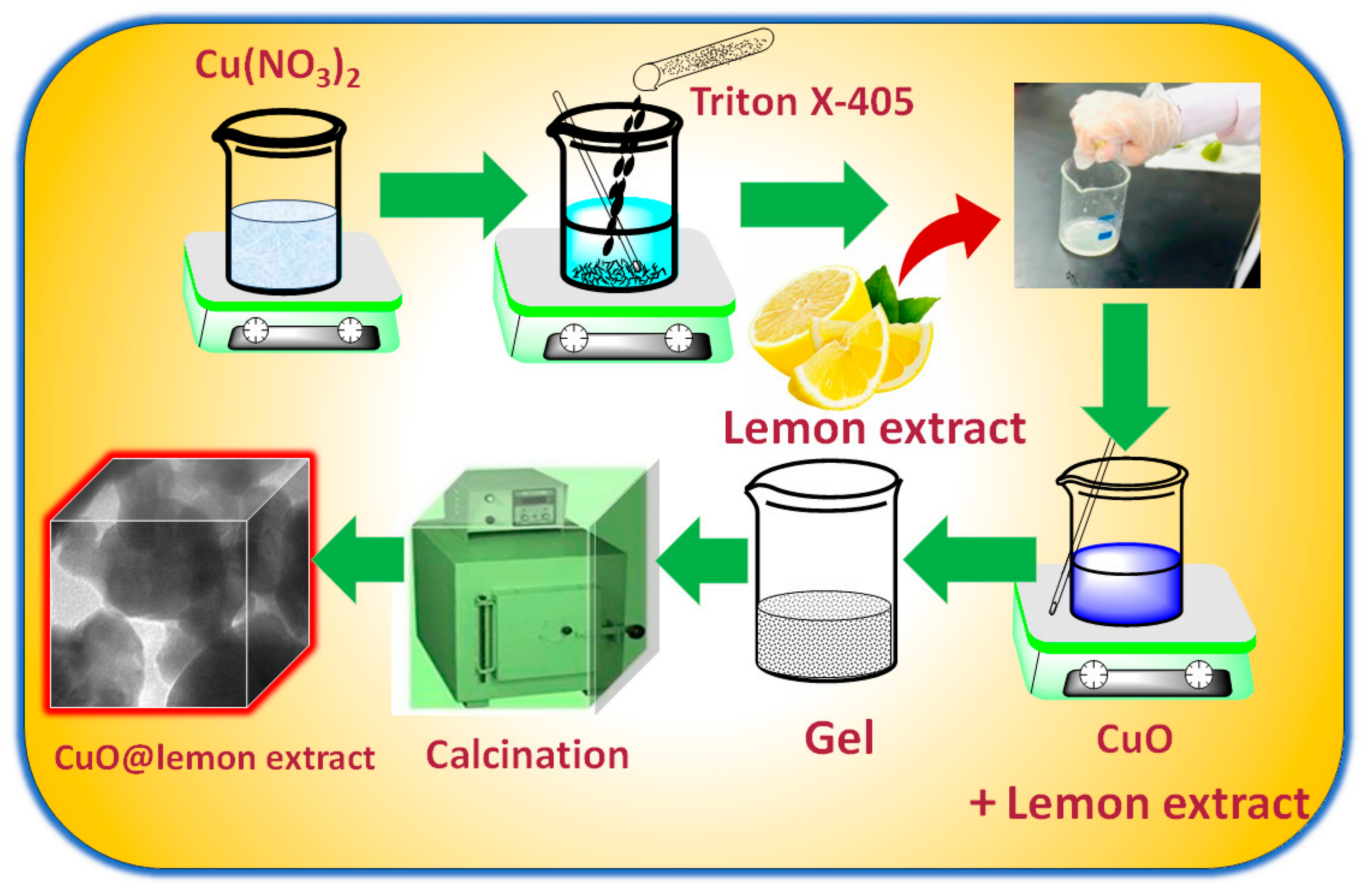
CuO@lemon extract synthesis diagram.

The CuO@lemon extract was synthesized by dissolving 2 g of Cu(NO_3_)_2_ (50 wt% from BDH-MERCK) in 2 g of high-purity water (50 wt%). A quantity of 4 g of triton X 405 (Sigma-Alrich-MilliporeSigma, Burlington, MA, USA, 70% wt%) was progressively added to this solution. The lemon extract utilized in our study was obtained from ripe Eureka lemons, which were procured from a local organic supplier. Lemon peels were thoroughly washed and subjected to a cold-pressing extraction method in which mechanical pressure was applied to release the essential-oil content from the peels. No pre-treatment steps were employed. This information has been incorporated to enhance the transparency of our methodology, ensuring that readers and researchers have a comprehensive understanding of our lemon-extract preparation procedure. A rich blue gel was produced after adding 4 mL fresh lemon extract and stirring the mixture for 30 min. The gel underwent a 5 h calcination treatment at 500 °C in a furnace with cooling and heating rates of 4.17 °C/min before it reached ambient temperature after being held at ambient temperature for 120 h. The defined CuO@lemon-extract nanoporous materials were submitted to a variety of analytical procedures before being used in glucose sensing (Scheme 1).

### 4.1. Preparation of Modified CuO@lemon-Extract-GCE Sensing Platform

The GCE surface (a glassy carbon electrode with a diameter of 3 mm would have a geometrical area of approximately 0.071 cm^2^) was subjected to modification by applying a dispersion of CuO@lemon-extract nanoporous materials. The dispersion was made by dissolving 5.0 mg of fabricated monoliths in 10 mL of ethanol ultrasonically. Prior to the electrode modification process, the GCE surface underwent polishing using alumina slurry, followed by cleaning through sonication in ethanol for 10 min. After that, the electrode was washed via distilled water and allowed to dry at the normal temperature. A 10 μL volume of the dispersed mixture was subsequently deposited onto the GCE surface, and the solvent was left to evaporate naturally. This modified electrode, referred to as CuO@lemon-extract GCE, depending on the specific material used, was utilized for subsequent electrochemical investigations [23].

### 4.2. Instrumentation

Powdered X-ray diffraction (P-XRD) analysis was performed utilizing Cu Ka radiation and the X Pert PRO X-ray diffraction equipment. Morphological evaluation, facilitated by SEM and EDX, was executed using the JEOL JSM-6510LA electron microscope. Detailed insights into nanoparticle structure, size distribution, and morphology were gained through TEM examination, employing the JEM1400 instrument. The synthesis process for all nanoporous materials was executed in a Muffle Furnace, specifically the HD150 PAD model. Electroanalytical investigations, including techniques such as cyclic voltammetry (CV), DPV, and amperometry, were conducted using the Autolab PGSTAT204 FRA32M instrument.

## 5. Conclusions

The formation of CuO@lemon-extract GCE signifies an important milestone in glucose sensing. The CuO@lemon-extract GCE produced has a consistent nanoparticle size, at 20 to 30 nm on a 50 nm scale. CuO@lemon-extract GCE demonstrated rapid electron transfer, a significant capability for measuring sensing of glucose to the lowest possible limit (0.01 μM), a sensitivity of 3293 μA mM^−1^ cm^−2^, and a broad linear range, from 0.01 μM to 0.2 μM. The electrode exhibits exceptional sensing capability, including ultrahigh sensitivity, a broad linear range, a fast reaction time, and great stability, reproducibility, and repeatability. This study involved the synthesis of CuO nanoparticles with lemon extract, which was subsequently deposited onto a GCE. The utilization of CuO@lemon-extract-modified GCE offers multiple advantages. The finely controlled electrochemical deposition process allows for the large-scale invention of consistent sensing of glucose interfaces. Moreover, this method is appropriate for the accurate and controlled alteration of nanosensors.

## Figures and Tables

**Table 1:** NEGS comparison table.

Electrode	Potential (V)	Sensitivity (µA mM^−1^ cm^−2^)	Linear Range	LOD (µM)	Ref.
CuO nanourchins	0.57	1634	5 mM	1.97	[23]
CuO nanospheres (NS)	0.57	730	6 mM	2.11	[24]
CuO nanobelts (NBs)	0.6	582	None	1.0	[25]
CuO NS	0.6	404.5	2.6 mM	1.0	[26]
CuO NPs	0.55	1397	2.3	0.5	[27]
CuO NWs	0.4	431	2.5 mM	0.8	[17]
Porous CuO	0.65	2900	2.5	0.14	[28]
CuO@CNTFs	0.6	~3000	13 mM	1.4	[29]
CuO/Ag/NiO	0.65	2895.3	0.001–5.50 mM	0.1	[6]
Mesoporous copper (I) oxide nanoparticles	-	733	1 to 9 mM	0.05	[30]
Cu_2_O/MXene graphene aerogel (3D) composite	-	264.5	0.1 mM–40 mM	1.1	[31]
CuO@lemon-extract GCE	0.53	3293	0.01–0.20 µM	0.01	This Work

## Data Availability

All data generated or analysed during this study are included in this published article.

## Sample Availability

The authors declare that the samples or materials used in this study are available upon request for research purposes.
